# Supplementary material for: The Use of Heterologous Antigens for Biopanning Enables the Selection of Broadly Neutralizing Nanobodies Against SARS-CoV-2
Source: Antibodies (Basel). 2025 Mar 7;14(1):23. doi: 10.3390/antib14010023 (PMC11939171; doi:10.3390/antib14010023)
Supplement: Supplementary file 1 [file antibodies-14-00023-s001.zip › antibodies-3477949-supplementary.pdf]

Supplementary materials

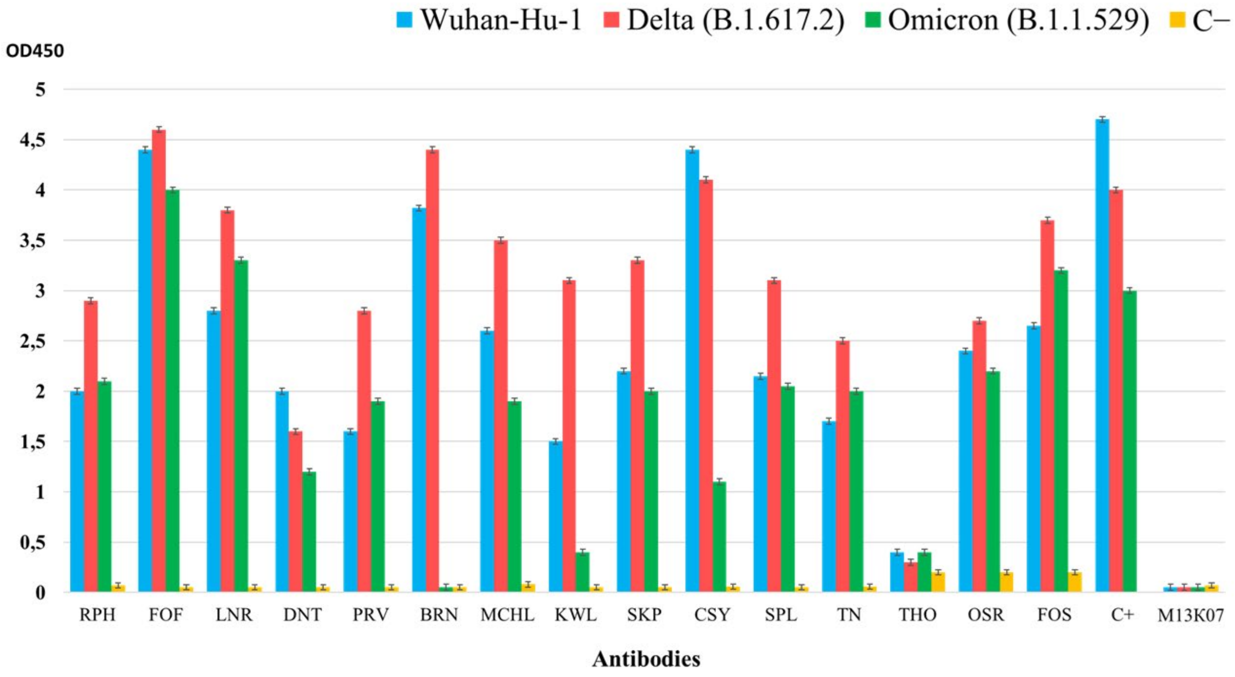

Figure S1. Evaluation of the binding of selected phage clones to SARS-CoV-2 S protein trimers after the third round of biopanning. A convalescent human serum was used as a positive control (C+), while recombinant domain III (DIII) of the West Nile virus was used as a negative control (C−). M13K07 helper phage served as a system negative control

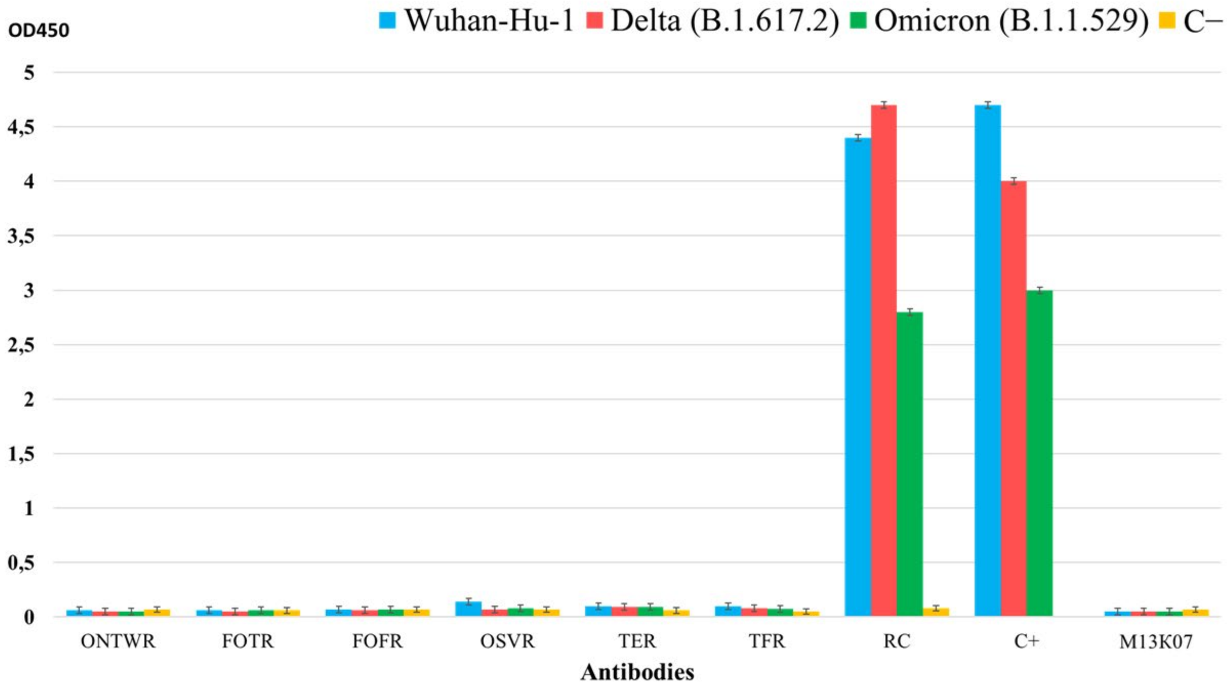

Figure S2. Evaluation of the binding of selected phage clones to SARS-CoV-2 S protein trimers after the third round of biopanning. A convalescent human serum was used as a positive control (C+), while recombinant domain III (DIII) of the West Nile virus was used as a negative control (C−). M13K07 helper phage served as a system negative control

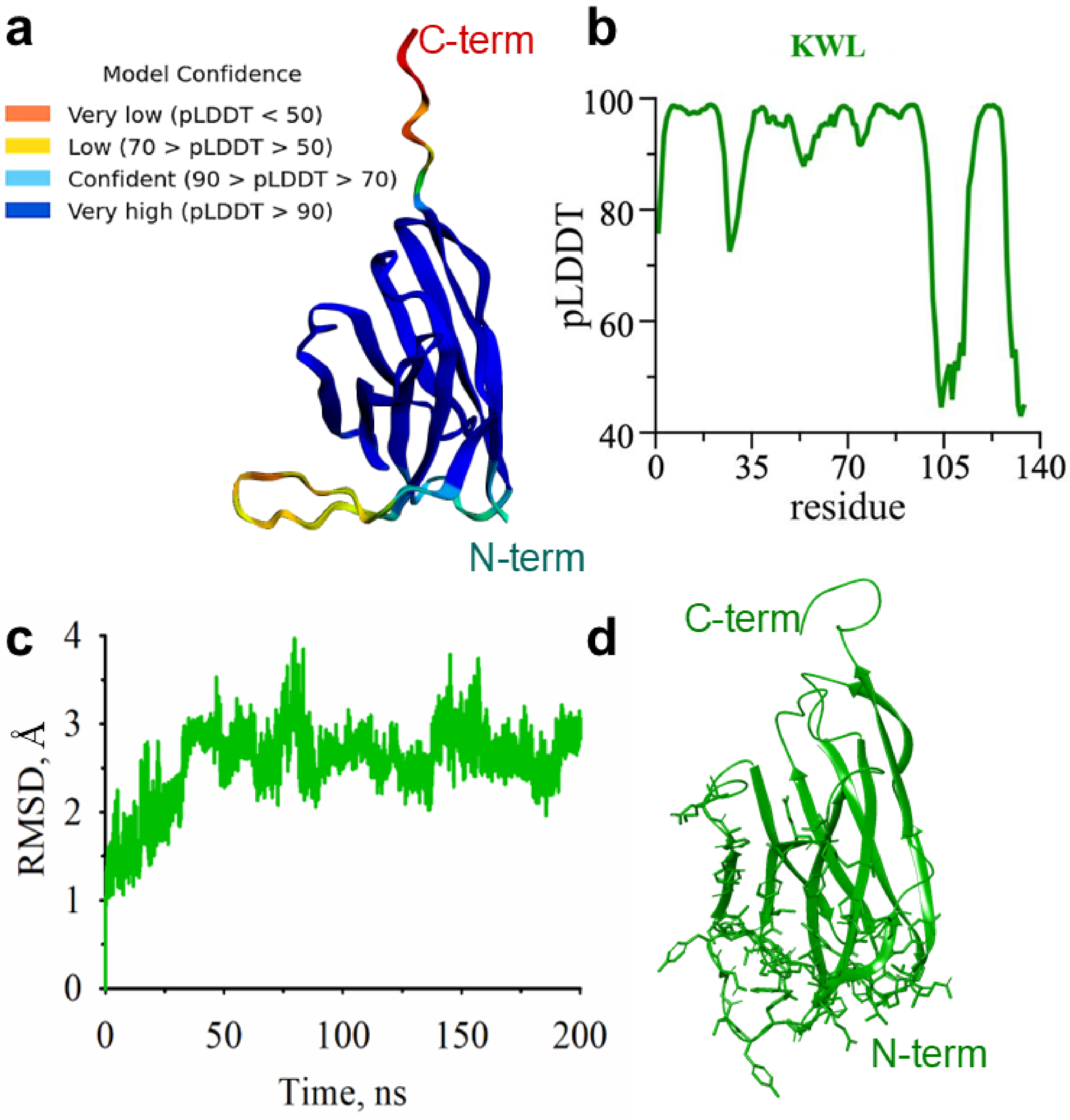

Figure S3. Theoretical calculation results: (a,b)—results of prediction procedure using AlphaFold2; pLDDT—predicted local distance difference test; (c)—the root mean square deviation (RMSD) is used to measure the average change in displacement of a selection of atoms for a particular frame with respect to a reference frame, calculated for all (1000) frames in the trajectory; (d)—the tertiary structure of the antibody KWL after the clustering procedure of frames of molecular dynamic simulations.

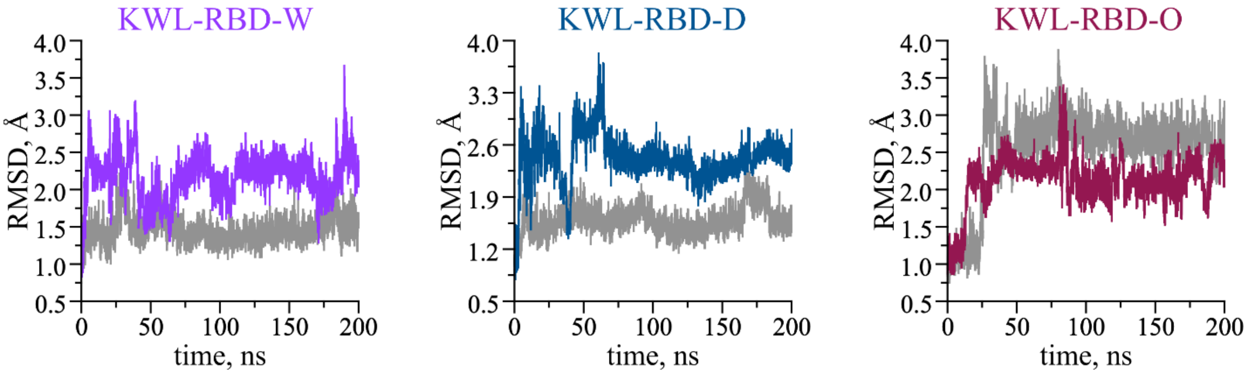

Figure S4. The root mean square deviation (RMSD) is used to measure the average change in displacement of a selection of atoms for a particular frame with respect to a reference frame. It is calculated for all (1000) frames in the trajectory

Table S1. Molecular (protein–protein) docking results

Docking pose	Contact amino acid residues		Number of intermolecular interactions				PIPER energy	PIPER score
	KWL	RBD-W	H-bond	Salt bridge	π-π stacking	Vdw (clash)		
1	17	21	3	1	0	46	−256.982	−320.671
2	16	28	2	0	0	39	−271.190	−174.106
7	20	18	2	3	0	31	−247.856	−103.162
14	17	19	3	0	0	52	−233.602	−284.089
16	23	21	2	0	2	32	−253.273	−26.051
19	20	26	1	1	0	90	−264.917	−102.899
30	19	21	2	1	0	47	−235.461	−93.638

Table S2. Molecular (protein–protein) docking results

Docking pose	Contact amino acid residues		Number of intermolecular interactions				PIPER energy	PIPER score
	KWL	RBD-D	H-bond	Salt bridge	π-π stacking	Vdw (clash)		
3	18	24	4	2	0	146	−235.005	−212.049
4	18	27	4	2	4	126	−253.550	−131.182
5	16	23	2	2	2	222	−251.837	−356.623
9	16	25	2	0	2	130	−233.705	−117.089
20	16	35	4	0	0	144	−234.899	−166.043
28	19	26	2	0	0	150	−237.535	−119.812
30	11	19	2	0	2	108	−236.608	−232.362

Table S3. Molecular (protein–protein) docking results

Docking pose	Contact amino acid residues		Number of intermolecular interactions				PIPER energy	PIPER score
	KWL	RBD-O	H-bond	Salt bridge	π-π stacking	Vdw (clash)		
1	12	17	1	1	1	26	−246.031	−185.632
3	10	17	0	0	1	27	−234.343	−223.673
8	18	23	0	0	0	46	−238.285	−217.087
9	15	19	3	0	1	29	−230.050	−219.127
14	12	17	2	2	1	32	−233.718	−241.470
15	12	15	0	1	1	40	−234.014	−110.889
28	19	22	3	0	0	48	−253.614	−198.956
